# The Effects of Bariatric Surgery on Renal, Neurological, and Ophthalmic Complications in Patients with Type 2 Diabetes: the Taiwan Diabesity Study

**DOI:** 10.1007/s11695-020-04859-9

**Published:** 2020-07-18

**Authors:** Yi-Cheng Chang, Seh-Huang Chao, Ching-Chu Chen, Kong-Han Ser, Keong Chong, Chieh-Hsiang Lu, Meng-Lun Hsieh, Yu-Yao Huang, Yi-Chih Lee, Chih-Cheng Hsu, Lee-Ming Chuang, Wei-Jei Lee

**Affiliations:** 1 Department of Internal Medicine, National Taiwan University Hospital, Taipei, Taiwan; 2 Graduate Institute of Medical Genomics and Proteomics, National Taiwan University, Taipei, Taiwan; 3 Institute of Biomedical Sciences, Academia Sinica, Taipei, Taiwan; 4 Division of General Surgery, Jen-Ai Hospital, Taichung, Taiwan; 5 Division of Endocrinology and Metabolism, Department of Medicine, China Medical University Hospital, Taichung, Taiwan; 6 School of Chinese Medicine, China Medical University, Taichung, Taiwan; 7 Department of Surgery, Ten-Chen General Hospital, Taoyuan, Taiwan; 8 Department of Medicine, Min-Sheng General Hospital, Taoyuan, Taiwan; 9 Division of Metabolism & Endocrinology, Chia-Yi Christian Hospital, Chia-Yi, Taiwan; 10 Division of Metabolism & Endocrinology, Chang Gung Memorial Hospital, Taoyuan, Taiwan; 11 Department of International Business, Chien Hsin University of Science and Technology, Taoyuan, Taiwan; 12 Institute of Population Health Sciences, National Health Research Institutes, Zhunan, Taiwan; 13 Graduate Institute of Molecular Medicine, National Taiwan University, Taipei, Taiwan; 14 Division of General Surgery, Min-Sheng General Hospital, Taoyuan, Taiwan; 15 Department of Surgery, National Taiwan University Hospital, Taipei, Taiwan

**Keywords:** Bariatric surgery, Diabetic neuropathy, Diabetic nephropathy, Diabetic opthalmopathy

## Abstract

**Background:**

Bariatric surgery has been shown to improve glycemic control in patients with type 2 diabetes. However, less is known whether it can also reduce diabetic renal, neurological, and ophthalmic complications.

**Methods:**

This prospective multicenter cohort study compared renal, ophthalmic, and neurological complications between 49 patients with obesity/overweight receiving bariatric surgery and 338 patients receiving standard medical treatment after follow-up for 2 years. Patients received neurological examinations including toe tuning fork vibration test, ankle tendon reflex test, 10-g monofilament test, and ophthalmic examinations including visual acuity measurement and fundus examinations. Multiple regressions, propensity score weighting, and matching were employed to adjust for baseline differences.

**Results:**

After 2 years of follow-up, patients with type 2 diabetes receiving bariatric surgery had greater reduction in BMI, HbA1c, and urine albumin–creatinine ratio, greater improvement in estimated glomerular filtration rate, and greater increase in tuning fork test score of right and left toes compared with the medical group. However, there is no improvement in 10 g-monofilament test, visual acuity, diabetic non-proliferative retinopathy, and proliferative retinopathy. Similar results were obtained using multiple regression adjustment, propensity-score weighting, or comparing age-, sex-, and BMI-matched subjects.

**Conclusions:**

After 2-year follow-up, patients with obesity/overweight and type 2 diabetes receiving bariatric surgery have increased glomerular filtration rate, reduced albuminuria, and improved tuning folk vibration sensation.

**Electronic supplementary material:**

The online version of this article (10.1007/s11695-020-04859-9) contains supplementary material, which is available to authorized users.

## Introduction

Type 2 diabetes mellitus and obesity, or termed diabesity, have reached epidemic proportions worldwide. Abundant evidence demonstrated that bariatric surgery prevents cardiovascular diseases in patients with obesity. Furthermore, bariatric surgery is very effective for glycemic control in patients with type 2 diabetes and a substantial proportion of patients with diabetes achieve complete remission after surgery. However, less is known about whether bariatric surgery can effectively reduce diabetic microvascular complications including nephropathy, neuropathy, and retinopathy in patients with diabetes.

The Swedish Obese Subjects (SOS) matched prospective cohort studies reported reduced cumulative incidence of microvascular complications in surgery patients compared with medical control patients [1]. However, diabetic microvascular complications in this study were defined by diagnostic codes through linking to the national registry database. A large retrospective observational cohort study using four health insurance databases in the USA also reported substantial reduction in cumulative incidence of diabetic neuropathy, nephropathy, and retinopathy in patients with obesity and diabetes receiving bariatric surgery [2]. Similarly, another large retrospective cohort study using health care administrative database in the USA showed substantially decreased microvascular complications in patients receiving bariatric surgery [3]. However, all these studies defined diabetic microvascular complications primarily through diagnostic codes but not clinical or laboratory examinations.

Here, we conducted a multicenter prospective cohort study, the Taiwan Diabesity Study, to investigate the effects of bariatric surgery on diabetic renal, neurological, and ophthalmic complications used on standardized physical examinations and laboratory tests.

## Methods

### Patient Recruitment

The Taiwan Diabesity Study is a prospective multicenter observational cohort study primarily aimed to investigate the end-organ damage of patients with overweight/obesity and type 2 diabetes. Patients receiving bariatric surgery including gastric bypass and sleeve gastrectomy were compared with controls receiving standard medical therapy. The study was conducted in six hospitals in Taiwan. All information regarding medical and surgical treatments was equally provided to each patient and the choice of treatment was made by the patients [4]. All sites had approval for the study from the respective institutional review boards. Informed consent was obtained from all participants. All procedures performed in studies involving human participants were in accordance with the Helsinki declaration. The details of the study design have been described in our previous study [4].

Inclusion criteria were as follows:

- Taiwan citizens, aged 20–67 years
- Type 2 diabetes treated in a diabetes center for more than 6 months
- BMI 25 kg/m^2^

Exclusion criteria were as follows:

- Cancer in the past 5 years
- Previous bariatric surgery
- Body mass index (BMI) > 60 kg/m^2^
- Significant T2DM complications including blindness, amputation (including any part of the body), chronic kidney disease (serum creatinine > 2 mg/ml), dialysis, major adverse cardiovascular events, and stroke
- Unstable psychiatric illnesses
- Refusal to give consent or adhere to follow-up

### Data Collection

Each patient received examination including height, weight, blood pressure measurement, standard ophthalmic examinations including visual acuity and fundus examination, and neurological examinations including inspection, toe tuning fork vibration test, ankle reflex tests, and 10-g monofilament test [5]. The examiner strokes a 128-Hz tuning fork and then placed the fork at the dorsum of the interphalangeal joint of the hallux. The tuning fork score ranged from 0 to 8 points with decreasing magnitude of vibration. If the patients perceive higher tuning fork vibration points, the patients were defined to have higher sensitivity to tuning fork vibration. The ankle tendon reflex score is defined as 0 if the tendon reflex is present, 0.5 if the tendon reflex is present after enhancement, and 1 if the tendon reflex is absent. The 10-g monofilament test was performed using a 10-g Semmes-Weinstein monofilament at 8 points in each foot, including the plantar surface of the 1st, 3rd, and 5th digits; the plantar aspect of the medial, central, and lateral aspect of the mid-foot; the posterior of the plantar foot; and the point between the first and second toes on the dorsal surface of the foot. The monofilament is applied perpendicularly to the skin surface. The examiner applied sufficient force to cause the filament to bend and asked the patient to respond blindly if the pressure is detected. The monofilament score is 0 if pressure is detected by the patient at all 8 points, 0.5 for 7 or fewer points detected, and 1 for non-detection of any point. The International Classification of Diabetic Retinopathy was used for funduscopic grading of diabetic retinopathy. This system classified diabetic retinopathy into five levels, including (1) no apparent retinopathy, (2) mild non-proliferative diabetic retinopathy (NPDR), (3) moderate NPDR, (4) severe NPDR, and (5) proliferative diabetic retinopathy. Owing to small sample size, mild, moderate, and severe NPDR were all classified as one category [6]. Diabetic neuropathy was graded by the Michigan Neuropathy Screening Instrument score with a cut-off value of 2.5 point [7, 8]. Laboratory measurements included fasting plasma glucose, hemoglobin A1c (HbA1c), high-density lipoprotein cholesterol (HDL-C), low-density lipoprotein cholesterol (LDL-C), triglycerides, creatinine levels, dipstick urine protein test, and urine albumin and creatinine. Estimated glomerular filtration rate (eGFR) were calculated by the Modification of Diet in Renal Disease study equation (MDRD eGFR) and the Chronic Kidney Disease Epidemiology Collaboration equation (CKD EPI eGFR).

### Statistical Analysis

All statistical analyses were performed using SAS version 8.0. Continuous variables with normal distribution were compared using Student’s *t* test of independent samples. Continuous variables with skewed distribution or ordinal variables including fasting plasma glucose, HbA1c, triglycerides, creatinine, albumin–creatinine ratio, tuning fork vibration test, ankle tendon reflex score, monofilament score, and visual acuity were compared using Wilcoxon rank-sum test.

To correct for baseline differences, multiple regression adjustments, propensity score weighting, and matching were performed. Multiple regressions were conducted using age, sex, baseline BMI, HbA1c, LDL-C, blood pressures, and duration of diabetes as covariates. For multiple regressions of creatinine, albumin–creatinine ratio, and eGFR, the use of GLP-1 agonists and angiotensin-converting-enzyme (ACE) inhibitors/angiotensin receptor blockers (ARB) were further added as covariates.

Propensity score weighting was employed to correct indication bias of receiving standard medical therapy and bariatric surgery. Matching procedure was conducted 1:1 fixed-ratio propensity score matching that employed a nearest-neighbor algorithm. Propensity scores were calculated using a logistic regression model in which the dependent variable was 1 for patients receiving standard medical treatment and 0 for those receiving bariatric surgery. The propensity score model was a non-parsimonious simple logistic regression with baseline covariates including age, sex, and baseline BMI. The estimated probability of receiving treatment, that is, the propensity score, was utilized to correct the differences between two treatment groups at the baseline.

## Results

### Participant Characteristics

The baseline characteristics of 338 patients with overweight/obesity and diabetes receiving standard medical therapy and 49 patients receiving bariatric surgery are listed in Table 1. Among 49 surgical patients, 22 received sleeve gastrectomy and 27 received Roux-en-Y gastric bypass surgery. At baseline, those who receiving bariatric surgery were younger, with a higher proportion of female patients, had larger waist circumference, higher BMI, serum LDL-C, serum triglycerides, and urine albumin–creatinine ratio, and faster pulse than those receiving standard medical treatment.

**Table 1:** Baseline characteristics

	Medical therapy		Bariatric surgery		*P* value
Number	338		49		
Variable	Mean	SD	Mean	SD	
Age (year)	51.12	9.64	44.94	11.01	**< 0.001**
Gender (male %)	62.72		40.82		**0.005**
Duration of diabetes (year)	8.72	5.55	7.02	4.81	**0.036**
Weight (kg)	81.94	12.52	95.88	20.63	**< 0.001**
Waist circumference (cm)	99.59	10.01	112.67	15.04	**< 0.001**
Body mass index (kg/m^2^)	30.02	3.93	36.11	6.90	**< 0.001**
HbA1c (%) *	7.91	1.40	8.80	1.53	**< 0.001**
Fasting glucose (mg/dL)*	155.91	47.13	181.67	63.17	**0.016**
LDL cholesterol (mg/dL)	95.95	25.39	106.98	35.52	**0.040**
HDL cholesterol (mg/dL)	41.60	8.93	40.86	9.39	0.59
Triglycerides (mg/dL)*	181.24	126.38	294.86	295.87	**< 0.001**
Systolic blood pressure (mmHg)	133.52	13.64	135.64	13.70	0.31
Diastolic blood pressure (mmHg)	83.23	10.19	83.04	7.64	0.88
Creatinine (mg/dL)*	0.87	0.22	0.87	0.31	0.18
Urine albumin–creatinine ratio (mg/g)*	119.0	415.0	538.8	2836.1	**0.001**
eGFR-MDRD (mL/min/1.73 m^2^)	88.69	20.94	87.95	22.41	0.82
eGFR-CKD EPI (mL/min/1.73 m^2^)	93.05	17.96	95.16	22.42	0.53
Pulse (/min)	81.26	10.94	88.24	11.27	**< 0.001**
Toe tuning fork score, right	6.87	1.01	7.01	0.84	0.50
Toe tuning fork score, left	6.84	0.97	7.05	0.83	0.32
Ankle reflex score, right (0, 0.5, 1)*	0.03	0.12	0.07	0.23	0.19
Ankle reflex score, left (0, 0.5, 1)*	0.03	0.12	0.07	0.23	0.19
10-g monofilament score, right*	0.01	0.05	0.00	0.00	0.44
10-g monofilament score, left*	0.00	0.05	0.00	0.00	0.51
Visual acuity, right*	0.77	0.27	0.76	0.34	0.47
Visual acuity, left*	0.79	0.26	0.81	0.32	0.14
Non-proliferative retinopathy, right	0.14	0.35	0.10	0.31	0.47
Non-proliferative retinopathy, left	0.13	0.34	0.10	0.31	0.58
Proliferative retinopathy, right	0.02	0.15	0.04	0.20	0.56
Proliferative retinopathy, left	0.02	0.15	0.04	0.20	0.56
All retinopathy	0.32	0.71	0.29	0.71	0.64
Metformin	0.85		0.20		**< 0.001**
Sulfonylurea	0.60		0.082		**< 0.001**
Glitazone	0.15		0.04		0.38
DPP4 inhibitor	0.44		0.082		**< 0.001**
Thiazolidinedione	0.12		0.0		**0.002**
GLP-1 analogues	0.023		0.0		0.36
α-glucosidase inhibitor	0.015		0.0		0.51
Insulin	0.22		0.061		**0.004**
ACE inhibitor /ARB	0.47		0.10		**< 0.001**
Other anti-hypertensives	0.27		0.20		0.32
Statins	0.56		0.24		**< 0.001**
Anti-platelet therapy	0.059		0.061		0.58

*Wilcoxon rank sum test

Toe tuning fork test, 0 to 8 points when the magnitude of vibrating fork decreased

Ankle reflex score 0, present; 0.5, present after enhancement; 1, absent

10-g monofilament score 0, present (> 8 points); 0.5, attenuated (1–7 points); 1, absent (0 point)

*HbA1c*, hemoglobin A1c; *LDL*, low-density lipoprotein; *HDL*, high-density cholesterol; *eGFR MDRD*, estimated glomerular filtration rate by the Modification of Diet in Renal Disease (MDRD) study equation

*CKD-EPI*, Chronic Kidney Disease Epidemiology Collaboration

*DPP4-inhbitors*, dipeptidyl peptidase 4; *GLP-1*, glucagon-like peptide 1; *ACE*, angiotensin-converting enzyme; ARB, angiotensin receptor blocker

All statistically non-significant values to non-bold and all statistically significant values to bold

#### Renal, Neurological, and Ophthalmological Outcomes Between Standard Medical Treatment Group and Bariatric Surgery Group

After follow-up for 2 years, those receiving bariatric surgery had significantly more weight loss, greater reduction of BMI, HbA1c, and triglycerides, greater increase in HDL-C, and greater decrease in systolic and diastolic blood pressure than the standard medical group (Tables 2 and 3). Similar results were obtained using baseline age-, sex-, and BMI-matched patients (Table 4).

**Table 2:** Changes after follow-up for 24 months adjusted by multiple regression

	Medical therapy		Bariatric surgery			
Number	338		49		*P*	P**
Variable	Mean	SD	Mean	SD		
Change of weight (kg)	− 0.78	5.23	− 24.50	17.31	**< 0.001**	**< 0.001**
Change of waist circumference (cm)	− 0.34	6.52	− 21.59	12.40	**< 0.001**	**< 0.001**
Change of Body mass index (kg/m^2^)	− 0.33	1.79	− 9.22	4.43	**< 0.001**	**< 0.001**
Change of HbA1c (%)*	− 0.35	1.24	− 2.88	1.52	**< 0.001**	**< 0.001**
Change of fasting glucose (mg/dL)*	− 10.75	55.82	− 75.57	66.70	**< 0.001**	**< 0.001**
Change of LDL cholesterol (mg/dL)	− 2.27	25.48	− 12.91	50.17	0.1	**0.013**
Change of HLD cholesterol (mg/dL)	1.83	20.75	11.49	11.05	**< 0.001**	**0.004**
Change of triglycerides (mg/dL)*	− 5.07	132.47	− 187.9	271.73	**< 0.001**	**< 0.001**
Change of systolic blood pressure (mmHg)	− 1.00	15.34	− 19.10	29.56	**< 0.001**	**< 0.001**
Change of diastolic blood pressure (mmHg)	− 3.99	10.32	− 12.78	17.50	**0.001**	**< 0.001**
Change of creatinine (mg/dL)*	0.54	6.64	− 0.07	0.23	**< 0.001**	0.94***
Change of albumin–creatinine ratio (mg/g)*	16.34	270.9	− 354.3	1923.5	**< 0.001**	0.002***
Change of eGFR-MDRD (mL/min/1.73 m^2^)	− 4.10	14.77	5.37	18.53	**0.001**	0.002***
Chang of eGFR-CKD EPI (mL/min/1.73 m^2^)	− 3.35	12.59	3.80	15.72	**0.004**	0.012***
Change of pulse (/min)	1.11	9.90	− 16.95	22.97	**< 0.001**	**< 0.001**
Change of toe tuning fork score, right*	− 0.04	0.82	0.84	0.042	**0.007**	**0.007**
Change of toe tuning fork score, left*	0.02	0.96	0.33	0.94	**0.016**	**0.013**
Change of ankle reflex score, right*	0.00	0.11	− 0.06	0.25	0.076	0.14
Change of ankle reflex score, left*	0.00	0.13	− 0.06	0.25	0.078	0.27
Change of monofilament score, right*	0.00	0.07	0.01	0.08	0.30	0.47
Change of monofilament score, left*	0.00	0.09	0.01	0.08	0.45	0.79
Change of visual acuity, right*	− 0.08	0.35	− 0.06	0.36	0.73	0.88
Change of visual acuity, left*	− 0.06	0.36	− 0.07	0.33	0.47	0.58
Change of non-proliferative retinopathy status, right (%)	0.08	0.50	0.08	0.57	0.99	0.53
Change of non-proliferative retinopathy status, left (%)	0.11	0.64	0.08	0.57	0.84	0.85
Change of proliferative retinopathy, left (%)	0.01	0.19	0.00	0.00	0.71	0.54
Change of proliferative retinopathy (%)	0.02	0.36	0.00	0.00	0.55	0.62
Metformin	0.038		0.0		0.43	
Sulfonylurea	0.0059		0.0		0.91	
Glitazone	0.021		− 0.041		**0.017**	
DPP4-inhibitor	− 0.03		0.0		0.66	
Thiazolidinedione	0.047		0.02		0.52	
GLP-1 analogues	0.015		0		0.84	
α-glucosidase inhibitor	− 0.006		0.02		0.051	
Insulin	− 0.012		− 0.041		0.41	
ACE inhibitor /ARB	0.02		− 0.02		0.47	
Other anti-hypertensives	0.030		− 0.12		**0.0042**	
Statins	0.11		− 0.04		**0.043**	
Anti-platelet therapy	0.0088		− 0.02		0.43	

*Wilcoxon rank-sum test

**adjusted for age, sex, body mass index, HbA1c, LDL-C, blood pressure, duration of diabetes

***adjusted for age, sex, body mass index, hemoglobin A1c, LDL-C, blood pressure, duration of diabetes, use of GLP-1 agonists and angiotensin-converting-enzyme inhibitors/angiotensin receptor blockers

Toe tuning fork test 0–8 points when the magnitude of vibrating fork decreased

Ankle reflex score 0, present; 0.5, present after enhancement; 1, absent

10-g monofilament score 0, present (> 8 points); 0.5, attenuated (1–7 points); 1, absent (0 point)

*HbA1c*, hemoglobin A1c; *LDL*, low-density lipoprotein; *HDL*, high-density cholesterol; *eGFR MDRD*, estimated glomerular filtration rate by the Modification of Diet in Renal Disease (MDRD) study equation; *CKD-EPI*, Chronic Kidney Disease Epidemiology Collaboration; *GLP-1*, glucagon-like peptide-1; *ACE*, angiotensin-converting-enzyme; *ARB*, angiotensin receptor blockers

All statistically non-significant values to non-bold and all statistically significant values to bold

**Table 3:** Changes after follow-up for 24 months weighted by propensity score

	Medical therapy		Bariatric surgery		*P* value
Number	338		49		
Variable	Mean	SD	Mean	SD	
Change of weight (kg)	− 2.37	2.64	− 30.66	8.76	**< 0.001**
Change of waist circumference (cm)	− 1.76	3.52	− 26.43	7.08	**< 0.001**
Change of body mass index (kg/m2)	− 0.89	0.93	− 11.64	2.43	**< 0.001**
Change of HbA1c (%)*	− 0.52	0.46	− 2.91	0.81	**< 0.001**
Change of fasting glucose (mg/dL)*	− 13.08	19.88	− 75.06	34.12	**< 0.001**
Change of LDL-C (mg/dL)	− 1.93	7.89	− 5.91	27.76	0.59
Change of HLD-C (mg/dL)	1.56	5.25	10.45	6.45	**< 0.001**
Change of triglycerides (mg/dL)*	− 4.20	45.35	− 184.41	156.38	**< 0.001**
Change of systemic blood pressure (mmHg)	− 0.79	4.66	− 18.82	17.07	**< 0.001**
Change of diastolic blood pressure (mmHg)	− 3.43	3.39	− 12.29	9.53	**0.001**
Change of creatinine (mg/dL)*	0.24	1.26	− 0.07	0.15	**< 0.001**
Change of albumin–creatinine ratio (mg/g)*	29.07	103.01	− 454.9	1163.6	**< 0.001**
Change of eGFR-MDRD (mL/min/1.73 m^2^)	− 5.45	5.36	3.59	11.09	**< 0.001**
Change of eGFR-CKD-EPI (mL/min/1.73 m^2^)	− 3.94	3.97	3.45	9.67	**0.007**
Change of pulse (/min)	1.67	3.45	− 17.39	12.37	**< 0.001**
Change of toe vibration fork score, right*	− 0.18	0.23	0.23	0.50	**0.041**
Change of toe vibration fork score, left*	− 0.14	0.25	0.22	0.61	0.069
Change of ankle reflex score, right*	− 0.01	0.06	− 0.08	0.18	0.076
Change of ankle reflex score, left*	− 0.02	0.07	− 0.08	0.18	0.078
Change of monofilament score, right*	0.00	0.02	0.02	0.05	0.30
Change of monofilament score, left*	0.00	0.03	0.02	0.05	0.45
Change of visual acuity, right*	− 0.05	0.11	0.01	0.22	0.73
Change of visual acuity, left*	− 0.02	0.11	− 0.04	0.18	0.47
Change of non-proliferative retinopathy status, right (%)	0.01	0.16	0.18	0.30	0.16
Change of non-proliferative retinopathy status, left (%)	0.06	0.18	0.18	0.30	0.31
Change of proliferative retinopathy, right (%)	0.04	0.08	0.00	0.00	0.050
Change of proliferative retinopathy, left (%)	0.03	0.08	0.00	0.00	0.071

*Wilcoxon rank sum test

Toe tuning fork test, 0–8 points when the magnitude of vibrating fork decreased

Ankle reflex score 0, present; 0.5, present after enhancement; 1, absent

10-g monofilament score 0, present (> 8 points); 0.5, attenuated (1–7 points); 1, absent (0 point)

*HbA1c*, hemoglobin A1c; *LDL-C*, low-density lipoprotein cholesterol; *HDL-C*, high-density lipoprotein cholesterol; *eGFR MDRD*, estimated glomerular filtration rate by the Modification of Diet in Renal Disease (MDRD) study equation

*CKD-EPI*, Chronic Kidney Disease Epidemiology Collaboration

All statistically non-significant values to non-bold and all statistically significant values to bold

**Table 4:** Changes after follow-up for 24 months using baseline age-, sex, and BMI-matched subjects

	Medical therapy		Bariatric surgery		*P* value
Number	125		43		
Variable	Mean	SD	Mean	SD	
Age (year)	46.01	10.29	45.98	11.10	0.987
Gender (male %)	44.00		46.51		0.859
BMI (kg/m^2^)	32.86	4.31	34.60	5.02	**0.030**
Change of weight (kg)	− 1.57	7.32	− 22.54	17.21	**< 0.001**
Change of waist circumference (cm)	− 0.94	8.59	− 20.51	11.51	**< 0.001**
Change of body mass index (kg/m^2^)	− 0.64	2.52	− 8.38	3.85	**< 0.001**
Change of HbA1c (%)*	− 0.52	1.44	− 2.76	1.47	**< 0.001**
Change of fasting glucose (mg/dL)*	− 19.58	68.67	− 76.13	68.84	**< 0.001**
Change of LDL-C (mg/dL)	− 4.34	29.09	− 13.90	49.78	0.244
Change of HLD-C (mg/dL)	0.34	7.35	12.38	10.31	**< 0.001**
Change of triglycerides (mg/dL)*	2.98	154.26	− 190.93	284.18	**< 0.001**
Change of systolic blood pressure (mmHg)	0.33	14.99	− 17.81	28.59	**< 0.001**
Change of diastolic blood pressure (mmHg)	− 4.09	11.40	− 12.49	17.21	**0.004**
Change of creatinine (mg/dL)*	0.04	0.14	− 0.08	0.24	**0.006**
Change of albumin–creatinine ratio (mg/g)*	53.89	395.42	− 363.93	1996.91	**< 0.001**
Change of eGFR-MDRD (mL/min/1.73 m^2^)	− 4.04	14.50	6.27	18.97	**0.002**
Change of eGFR-CKD-EPI (mL/min/1.73 m^2^)	− 2.97	10.56	4.32	16.37	**0.009**
Change of pulse (/min)	1.40	10.20	− 16.67	23.15	**< 0.001**
Change of toe tuning fork score, right	− 0.21	0.67	0.37	0.81	**0.001**
Change of toe tuning fork score, left	− 0.15	0.71	0.43	0.92	**0.002**
Change of ankle reflex score, right*	0.00	0.16	− 0.08	0.24	0.081
Change of ankle reflex score, left*	− 0.01	0.18	− 0.08	0.24	0.086
Change of monofilament score, right*	0.00	0.08	0.01	0.08	0.39
Change of monofilament score, left*	0.00	0.08	0.01	0.08	0.39
Change of visual acuity, right*	− 0.09	0.35	− 0.06	0.37	0.69
Change of visual acuity, left*	− 0.05	0.35	− 0.07	0.34	0.77
Change of non-proliferative retinopathy status, right (%)	− 0.04	0.50	0.04	0.56	0.50
Change of non-proliferative retinopathy status, left (%)	0.06	0.52	0.04	0.56	0.87
Change of proliferative retinopathy, right (%)	0.03	0.24	0.00	0.00	0.32
Change of proliferative retinopathy, left (%)	0.03	0.24	0.00	0.00	0.32

*Wilcoxon rank sum test

Toe tuning fork test, 0–8 points when the magnitude of vibrating fork decreased

Ankle reflex score 0, present; 0.5, present after enhancement; 1, absent

10-g monofilament score 0, present (> 8 points); 0.5, attenuated (1–7 points); 1, absent (0 point)

*HbA1c*, hemoglobin A1c; *LDL*, low-density lipoprotein cholesterol; *HDL*, high-density lipoprotein cholesterol; *eGFR MDRD*, estimated glomerular filtration rate by the Modification of Diet in Renal Disease (MDRD) study equation; *CKD-EPI*, Chronic Kidney Disease Epidemiology Collaboration

All statistically non-significant values to non-bold and all statistically significant values to bold

For renal outcome, the surgical group had significantly greater reduction in urine albumin–creatinine ratio (− 354.3 ± 1923.5 vs. 16.34 ± 270.9 mg/g, crude *P* < 0.001) and greater improvement in eGFR-MDRD (5.37 ± 18.53 vs. − 4.10 ± 14.77 mL/min/1.73 m^2^, crude *P* = 0.001) and eGFR-CKD-EPI (3.80 ± 15.72 vs. − 3.35 ± 12.59 mL/min/1.73 m^2^, crude *P* = 0.004) than the medical group (Table 2). The results obtained using multiple regression-adjustment (Table 2), propensity score weighting (Table 3), and age-, sex-, and BMI-matched subjects were similar (Table 4).

For neurological outcome, the surgical group had higher right toe tuning fork vibration score (0.84 vs. − 0.04, crude *P* < 0.001) and left toe tuning fork vibration score (0.02 ± 0.96 vs. 0.33 ± 0.94 points, crude *P* = 0.016) compared with the medical group after 2-year follow-up. The results obtained using multiple regression-adjustment (Table 2), propensity score weighting (Table 3), and age-, sex-, and BMI-matched subjects were similar (Table 4). There was a trend of insignificant improvement in right ankle tendon reflex score (− 0.06 ± 0.25 vs. 0.00 ± 0.11 points, crude *P* = 0.076) and left ankle reflex score (0.33 ± 0.94 vs. 0.02 ± 0.96, crude *P* = 0.016) in the surgical group compared with the medical group (Table 2). Similar results were obtained using multiple regression-adjustment (Table 2), propensity score weighting (Table 3), and age-, sex-, and BMI-matched subjects (Table 4). However, there was no improvement in 10-g monofilament score bilaterally (Tables 2, 3, and 4).

For ophthalmic outcome, there was no improvement in visual acuity, diabetic non-proliferative retinopathy, and proliferative retinopathy bilaterally (Tables 2, 3, and 4).

Subgroup analysis was then performed in patients with established and non-established complications of diabetes. Owing to small sample size, subgroup analysis was performed only in patients with or without microalbuminuria (Supplementary Tables 1 and 2) and diabetic neuropathy (Supplementary Tables 3 and 4) at baseline. The results were similar except that improvement in toe tuning fork vibration and tendon reflex score is attenuated in patients with urine albumin–creatinine > 30 mg/g and those with Michigan Neuropathy Screening Instrument Score **>** 2.5 points at baseline.

## Discussion

This multicenter prospective cohort study found that patients with overweight/obesity and diabetes receiving bariatric surgery had greater reduction of albuminuria and greater improvement in eGFR and toe tuning fork vibration sensation compared with those receiving standard medical treatment. However, there is no improvement in ophthalmic complications in the surgical group compared with the medical group.

### Effects of Bariatric Surgery on Diabetic Albuminuria

This study also showed reduced albuminuria in the surgical group compared with the medical group. Such finding is consistent with results of most previous studies. A meta-analysis including 7 observational studies on 446 participants showed that patients with obesity and diabetes receiving bariatric surgery had significant reduction in albuminuria after surgery [9]. Importantly, in the randomized clinical trial, the Surgical Treatment and Medications Potentially Eradicate Diabetes Efficiency (STAMPEDE) study, patients with diabetes receiving bariatric surgery had reduced urine albumin–creatinine ratio of − 3.0 mg/g in the gastric bypass group and − 5.0 mg/g in the sleeve gastrectomy group compared with − 1.0 mg/g in the medical group (*P* = 0.03 and *P* = 0.002 respectively) [10]. Similar results were observed after 5-year follow-up of the study [11]. In another randomized trial involving 39 surgical patients and 15 medical patients, none of the subjects in the surgical group while 27% of subjects in the medical group had urine albumin–creatinine ratio > 30 mg/g after 5 years of follow-up [12]. Another smaller randomized clinical trial involving 19 patients with obesity and diabetes and 19 medical controls showed insignificant reduction of urine albumin–creatinine ratio (− 1.0 vs. 0.0 mg/g, *P* = 0.96) [13]. An observational study of 101 patients with obesity and diabetes showed decrease in urine albumin–creatinine ratio from a median of 80–30 mg/g after bariatric surgery [14]. Collectively, randomized clinical trials and observational studies consistently demonstrated reduced albuminuria in patients with diabetes after bariatric surgery.

### Effects of Bariatric Surgery on eGFR in Patients with Obesity and Patients with CKD

In addition, the surgical group had significant improvement in eGFR compared with the medical controls. In the literature, different effects of bariatric surgery on eGFR in patients with diabetes and those with chronic kidney disease (CKD) have been reported. Abundant evidence showed that patients with diabetes with glomerular hyperfiltration (eGFR > 125 min/ml/1.73m^2^) before bariatric surgery had decreased eGFR after surgery. In contrast, patients with CKD tended to have increased eGFR after bariatric surgery [15–19]. Decreased hyperfiltration in patients with obesity receiving bariatric surgery may be attributed to reduction in blood volume, sympathetic tone, and blood pressure, while increased eGFR in patients with CKD may be related to resolution of CKD risk factors, including hypertension, hyperglycemia, and obesity, after bariatric surgery.

### Effects of Bariatric Surgery on eGFR in Patients with Diabetes

Most previous studies investigating the effects of bariatric surgery on GFR in patients with diabetes had small sample size. A randomized trial involving 19 surgical patients and 19 medical controls showed that surgical patients had significantly reduced serum creatinine level compared with medical controls (− 0.07 vs. 0 mg/dL, *P* = 0.01) [13]. A longitudinal 10-year follow-up study comparing 22 patients receiving biliopancreatic diversion with 28 medical patients showed change in eGFR of 13.6% in the surgical treatment arm and − 45.7% in the medical treatment arm (*P* < 0.001) [20]. Another observational study involving 714 surgical patients and 714 medical controls with 62.4% of participants having diabetes mellitus showed that surgical patients had 9.84 mL/min/1.73 m^2^ greater GFR than controls after 3 years follow-up [21]. An observational cohort study involving 163 patients with obesity and diabetes receiving bariatric patients and 225 medical controls reported eGFR of 88.8 mL/min/1.73m^2^ in the surgical group and 81.0 mL/min/1.73m^2^ in the medical group (*P* = 0.001) after 3 years follow-up [22]. Another observational study involving 986 surgical patients and 985 matched medical controls with ~ 40% of participants having diabetes mellitus reported that surgical patients had 58% lower risk for an eGFR decline of > 30% and 57% lower risk of doubling of serum creatinine or ESRD after 3.8 years follow-up [20]. Subgroup analysis in patients with diabetes yielded similar results [23].

In contrast, in the STAMEDE clinical trial, the surgical group showed a trend of insignificant reduction in eGFR compared with the medical group after 5 years follow-up (− 1.1% in medical group, − 7.7% in gastric bypass group, − 6.2% in sleeve gastrectomy group). However, it should be noted that the baseline eGFR is also high in the study (106, 110, and 109 mL/min/1.73m^2^, respectively) [10]. An observational study involving 30 adolescents with obesity and diabetes and 63 medical controls showed significantly higher eGFR in the medical group compared with the surgical group after follow-up of 5 years [24]. Nevertheless, the baseline eGFR of both arms were equally high (118 vs. 115 mL/min/1.73m^2^). In this present study, the baseline eGFR was slightly lower in the medical group (88.69 mL/min/1.73m^2^) compared with the surgical group (87.95 mL/min/1.73m^2^). Taken together, results obtained showed that bariatric surgery could reduce glomerular hyperfiltration in patients having diabetes with high baseline eGFR but preserves eGFR in those with low baseline GFR.

### Effects of Bariatric Surgery on Diabetic Neuropathy

The surgical group showed significant improvement in toe vibration sensation compared with medical controls. The toe tuning fork vibration sensation test is a very sensitive and reproducible for detecting diabetic polyneuropathy [25]. Toe vibration is sensed by Pacinian corpuscles and is transmitted by the large myelinated fiber. Past studies investigating the effect of bariatric surgery on diabetic neuropathy are scarce. The DiaSurg1 observational study involving 20 patients with diabetes reported complete reversal of symptomatic neuropathy in 67% of patients. After bariatric surgery, the questionnaire-based Neuropathy Symptom Score (NSS) decreased from a median of 8–0 points, while the test-based Neuropathy Deficit Score (NDS) decreased from a median of 6–4 points. Interestingly, among the tests conducted in NDS, both toe vibration test and tendon reflex improved but the pin-prick test and temperature sensation test showed no improvement [26], suggesting beneficial effect only on large fiber but not small fiber. However, the present study observed no improvement in 10-g monofilament test, which is sensed by the Ruffini corpuscles and transmitted by the large fiber. It is not known why the 10-g monofilament test did not improve after surgery.

### Effects of Bariatric Surgery on Diabetic Retinopathy

Present findings showed no improvement in visual acuity and diabetic non-proliferative and proliferative retinopathy in the surgical group compared with medical controls after 2 years of follow-up. Similarly, the STAMPEDE clinical trial reported no change in retinopathy and visual acuity after 2 years of follow-up [27]. The results at 5-year follow-up of the STAMPEDE trial also found no difference in retinopathy between surgical and medical groups [11]. An observational study of 102 patients with obesity and diabetes showed that bariatric surgery did not prevent progression of diabetic retinopathy [28].

On the other hand, a meta-analysis of 4 observation studies involving 255 surgical patients and 171 medical controls showed reduced diabetic retinopathy in the surgical group (OR = 0.47, 95% 0.22–0.99) [29] . Another meta-analysis of 6 studies showed lower incidence of new retinopathy in the surgical group than the medical control group (HR 0.39, 95% 0.21–0.71) [30]. A large matched cohort study using 4 health insurance databases involving 4024 surgical patients and 11,059 medical controls reported lower incidence of diabetic retinopathy at 5 years (hazard ratio 0.55 [0.42–0.73] [2]. Another large matched cohort study using the national patient registry involving 1111 surgical patients and 1074 matched controls also reported reduced risk of diabetic retinopathy after a follow-up of 5.3 years (HR 0.52, 95% 0.39–0.69) [2]. Taken together, current evidence regarding effects of bariatric surgery on diabetic retinopathy is inconsistent.

The low percentage in the use of metformin, ACE inhibitors/ARB, and other anti-diabetic agents in the surgical group is counterintuitive. Possible causes included sampling bias due to small sample size and contraindication of metformin and ACE inhibitor/ARB in pregnancy because the surgical group consisted primarily of young females. In addition, there is a tendency of polypharmacy among the elderly in both Taiwan and Japan [31, 32], which may explain the lower percentage in use of anti-diabetics and anti-hypertensives in the surgical group, which is much younger than the medical group.

The unique strength of this study is the evaluation of diabetic complications using standard laboratory tests and not diagnostic codes only. The present study is the largest investigation demonstrating beneficial effects of bariatric surgery on diabetic neuropathy evaluated using standard laboratory tests.

The present study has several limitations. First, this is an observational study and not a randomized clinical trial. The baseline differences were respectively adjusted using multiple regression, propensity score weighting, and matching but with similar results obtained. Second, we did not incorporate the pin-prick test for evaluation of small-fiber function. Only large-fiber function was evaluated. Third, the follow-up period was only 2 years and longer observation period is needed. The sample size of this study is small, especially the surgical group. This is because most patients with type 2 diabetes qualified for metabolic surgery were not willing to receive surgery and some of them could not afford the co-payment. In addition, funduscopy and neurological examinations were not performed by the same person because of multicenter design, which may generate interpersonal variability. However, the report format is uniform and the procedures were performed and read by experienced diabetes educators and diabetologists. Furthermore, patients with major systemic diseases were excluded at entry; hence, the present findings cannot be extrapolated to all patients. Lastly, nerve conduction velocity (NCV) study, previously viewed as gold standard for the diagnosis of diabetic neuropathy, was not performed. However, the most common form of diabetic neuropathy, distal symmetric polyneuropathy (DSPN), predominantly affects small nerve fibers [33–35]. Nevertheless, NCV detects mainly abnormality of large but not small nerve fiber. The American Diabetes Association (ADA) recommends diabetic neuropathy screening including at least two assessments (128-Hz tuning fork, 10-g monofilament, tendon reflex, or pin-prick test) [35]. Combinations of more than one test have more than 87% sensitivity in detecting diabetic neuropathy. A longitudinal study showed that these simple tests are good predictors of risk for foot ulcer. Furthermore, a study that demonstrated the single use of 128-Hz tuning fork yielded results similar to the extended score of the International Consensus on the Diabetic Foot (ICDF) [25]. The ADA consensus for diabetic neuropathy recommended that NCV test is rarely needed except for atypical features [35]. Another study showed that NCV did not have better sensitivity and specificity in detecting diabetic neuropathy compared with toe fork vibration study or 10-g monofilament test [36].

In conclusion, this multicenter prospective study found that bariatric surgery reduces albuminuria, improves renal function, and enhances toe vibration sensation in patients with overweight/obesity and diabetes.

## Electronic supplementary material

ESM 1 (DOCX 35 kb)
