# Supplementary material for: The Effects of Bariatric Surgery on Renal, Neurological, and Ophthalmic Complications in Patients with Type 2 Diabetes: the Taiwan Diabesity Study
Source: Obes Surg. 2020 Jul 18;31(1):117–26. doi: 10.1007/s11695-020-04859-9 (PMC7808993; doi:10.1007/s11695-020-04859-9)
Supplement: Supplementary file 1 — (DOCX 35 kb) [file 11695_2020_4859_MOESM1_ESM.docx]

**Supplementary Table 1. Changes after follow-up for 24 months adjusted by multiple rin patients with urine albumin-creatinine ratio ≦ 30mg/g**

|  | Medical Therapy | | Bariatric surgery | |  |  |
| --- | --- | --- | --- | --- | --- | --- |
| Number | 234 | | 26 | | P | P** |
| Variable | Mean | StdDev | Mean | StdDev |  |  |
| Change of weight (kg) | -0.59 | 3.69 | -23.59 | 20.44 | **<0.001** | **<0.001** |
| Change of waist circumference (cm) | 0.49 | 4.02 | -20.93 | 11.21 | **<0.001** | **<0.001** |
| Change of Body mass index (kg/m^2^) | -0.26 | 1.18 | -9.41 | 3.21 | **<0.001** | **<0.001** |
| Change of HbA1c (%)* | -0.26 | 1.13 | -2.87 | 1.38 | **<0.001** | **<0.001** |
| Change of fasting glucose (mg/dL)* | -5.35 | 53.19 | -58.52 | 59.32 | **<0.001** | **0.002** |
| Change of LDL-Cholesterol (mg/dL) | -1.59 | 24.87 | -22.42 | 48.93 | **0.050** | **0.043** |
| Change of HLD-Cholesterol (mg/dL) | 2.09 | 24.10 | 11.83 | 9.31 | **<0.001** | **0.036** |
| Change of triglycerides (mg/dL)* | 5.19 | 132.53 | -144.56 | 170.25 | **<0.001** | **<0.001** |
| Change of systolic blood pressure (mmHg) | -0.57 | 14.51 | -15.38 | 26.18 | **0.009** | **<0.001** |
| Change of diastolic blood pressure (mmHg) | -3.46 | 10.10 | -11.60 | 14.28 | **0.009** | **<0.001** |
| Change of creatinine (mg/dL)* | 0.76 | 7.97 | -0.04 | 0.11 | 0.127 | 0.888 |
| Change of albumin-creatinine ratio (mg/g)* | 21.87 | 193.31 | 15.91 | 72.97 | **0.013** | 0.646 |
| Change of eGFR-MDRD(mL/min/1.73 m^2^) | -3.33 | 13.54 | 3.39 | 9.99 | **0.017** | 0.052 |
| Chang of eGFR-CKD EPI (mL/min/1.73 m^2^) | -3.83 | 15.92 | 5.67 | 14.72 | **0.005** | **0.014** |
| Change of pulse (/min) | 1.67 | 9.64 | -12.61 | 15.76 | **<0.001** | **<0.001** |
| Change of toe tuning fork score, right* | -0.03 | 0.79 | 0.41 | 0.90 | 0.064 | **0.008** |
| Change of toe tuning fork score, left* | -0.01 | 0.79 | 0.53 | 1.13 | 0.077 | **0.003** |
| Change of ankle reflex score, right* | 0.01 | 0.10 | -0.07 | 0.23 | 0.152 | **0.013** |
| Change of ankle reflex score, left* | 0.01 | 0.10 | -0.07 | 0.23 | 0.152 | **0.013** |
| Change of monofilament score, right* | 0.00 | 0.07 | 0.00 | 0.00 | 0.839 | 0.963 |
| Change of monofilament score, left* | 0.01 | 0.11 | 0.00 | 0.00 | 0.712 | 0.850 |
| Change of visual acuity, right* | -0.07 | 0.35 | 0.01 | 0.37 | 0.896 | 0.779 |
| Change of visual acuity, left* | -0.04 | 0.35 | -0.03 | 0.40 | 0.853 | 0.802 |
| Change of non-proliferative retinopathy status, right (%) | 0.08 | 0.51 | 0.08 | 0.49 | 0.972 | 0.851 |
| Change of non-proliferative retinopathy status, left (%) | 0.08 | 0.70 | 0.08 | 0.49 | 0.974 | 0.878 |
| Change of proliferative retinopathy, left (%) | 0.02 | 0.18 | 0.00 | 0.00 | 0.319 | 0.739 |
| Change of proliferative retinopathy (%) | 0.01 | 0.20 | 0.00 | 0.00 | 0.657 | 0.805 |

* Wilcoxon rank-sum test;

** adjusted for age, sex, body mass index, HbA1c, LDL-C, blood pressure, duration of diabetes

Toe tuning fork test: 0 to 8 points when the magnitude of vibrating fork decreased

Ankle reflex score: 0: present; 0.5: present after enhancement; 1: absent

10-g monofilament score: 0: present **(**> 8 points **)**; 0.5: attenuated (1-7 points); 1: absent(0 point)

HbA1c: hemoglobin A1c; LDL: low-density lipoprotein cholesterol; HDL: high-density lipoprotein cholesterol; eGFR MDRD estimated glomerular filtration rate by the Modification of Diet in Renal Disease (MDRD) Study equation; CKD-EPI: Chronic Kidney Disease Epidemiology Collaboration;

**Supplement Table 2. Changes after follow-up for 24 months adjusted by multiple regression**

**in patients with urine albumin creatinine ratio > 30mg/g**

|  | Medical Therapy | | Bariatric surgery | |  |  |
| --- | --- | --- | --- | --- | --- | --- |
| Number | 104 | | 23 | | P | P** |
| Variable | Mean | StdDev | Mean | StdDev |  |  |
| Change of weight (kg) | -1.21 | 7.64 | -25.52 | 13.30 | **<0.001** | **<0.001** |
| Change of waist circumference (cm) | -1.86 | 9.37 | -22.29 | 13.78 | **<0.001** | **<0.001** |
| Change of Body mass index (kg/m^2^) | -0.47 | 2.70 | -9.01 | 5.56 | **<0.001** | **<0.001** |
| Change of HbA1c (%)* | -0.56 | 1.44 | -2.89 | 1.70 | **<0.001** | **<0.001** |
| Change of fasting glucose (mg/dL)* | -22.89 | 59.83 | -95.86 | 70.64 | **<0.001** | **<0.001** |
| Change of LDL-Cholesterol (mg/dL) | -3.79 | 26.87 | -3.00 | 50.58 | **0.942** | 0.523 |
| Change of HLD-Cholesterol (mg/dL) | 1.17 | 7.79 | 11.13 | 12.83 | **0.001** | **<0.001** |
| Change of triglycerides (mg/dL)* | -28.19 | 130.04 | -235.09 | 348.80 | **<0.001** | **<0.001** |
| Change of systolic blood pressure (mmHg) | -1.96 | 17.08 | -23.29 | 33.06 | **0.006** | **0.001** |
| Change of diastolic blood pressure (mmHg) | -5.21 | 10.74 | -14.12 | 20.81 | **0.057** | 0.140 |
| Change of creatinine (mg/dL)* | 0.06 | 0.15 | -0.10 | 0.32 | **0.030** | **0.027** |
| Change of albumin-creatinine ratio (mg/g)* | 5.18 | 383.63 | -724.50 | 2700.36 | **0.001** | **0.019** |
| Change of eGFR-MDRD(mL/min/1.73 m^2^) | -3.41 | 10.13 | 4.25 | 20.46 | 0.093 | 0.130 |
| Chang of eGFR-CKD EPI (mL/min/1.73 m^2^) | -4.73 | 11.79 | 5.04 | 22.30 | 0.052 | 0.059 |
| Change of pulse (/min) | 0.03 | 10.39 | -21.67 | 28.49 | **0.002** | **0.002** |
| Change of toe tuning fork score, right* | -0.05 | 0.88 | 0.15 | 0.79 | 0.284 | 0.245 |
| Change of toe tuning fork score, left* | 0.07 | 1.22 | 0.15 | 0.70 | 0.419 | 0.444 |
| Change of ankle reflex score, right* | -0.01 | 0.14 | -0.05 | 0.28 | 0.733 | 0.706 |
| Change of ankle reflex score, left* | -0.02 | 0.17 | -0.05 | 0.28 | 0.746 | 0.440 |
| Change of monofilament score, right* | -0.01 | 0.06 | 0.02 | 0.11 | 0.094 | 0.090 |
| Change of monofilament score, left* | -0.01 | 0.06 | 0.02 | 0.11 | 0.093 | 0.086 |
| Change of visual acuity, right* | -0.11 | 0.37 | -0.14 | 0.33 | 0.876 | 0.734 |
| Change of visual acuity, left* | -0.09 | 0.39 | -0.12 | 0.24 | 0.616 | 0.655 |
| Change of non-proliferative retinopathy status, right (%) | 0.07 | 0.49 | 0.07 | 0.62 | 0.986 | 0.531 |
| Change of non-proliferative retinopathy status, left (%) | 0.13 | 0.54 | 0.00 | 0.68 | 0.410 | 0.839 |
| Change of proliferative retinopathy, left (%) | 0.00 | 0.17 | 0.00 | 0.00 | 1.000 | 0.547 |
| Change of proliferative retinopathy (%) | 0.00 | 0.35 | 0.00 | 0.00 | 1.000 | 0.547 |

* Wilcoxon rank-sum test;

** adjusted for age, sex, body mass index, HbA1c, LDL-C, blood pressure, duration of diabetes

Toe tuning fork test: 0 to 8 points when the magnitude of vibrating fork decreased

Ankle reflex score: 0: present; 0.5: present after enhancement; 1: absent

10-g monofilament score: 0: present **(**> 8 points **)**; 0.5: attenuated (1-7 points); 1: absent(0 point)

HbA1c: hemoglobin A1c; LDL: low-density lipoprotein cholesterol; HDL: high-density lipoprotein cholesterol; eGFR MDRD estimated glomerular filtration rate by the Modification of Diet in Renal Disease (MDRD) Study equation; CKD-EPI: Chronic Kidney Disease Epidemiology Collaboration;

**Supplement Table 3. Changes after follow-up for 24 months adjusted by multiple regression**

**in patients with Michigan Neuropathy Screening Instrument Score ≦2.5 points**

|  | Medical Therapy | | Bariatric surgery | |  |  |
| --- | --- | --- | --- | --- | --- | --- |
| Number | 296 | | 37 | | P | P** |
| Variable | Mean | StdDev | Mean | StdDev |  |  |
| Change of weight (kg) | -0.65 | 4.47 | -21.99 | 18.53 | **<0.001** | **<0.001** |
| Change of waist circumference (cm) | 0.05 | 5.29 | -20.16 | 12.82 | **<0.001** | **<0.001** |
| Change of Body mass index (kg/m^2^) | -0.30 | 1.53 | -8.43 | 4.31 | **<0.001** | **<0.001** |
| Change of HbA1c (%)* | -0.34 | 1.17 | -2.90 | 1.57 | **<0.001** | **<0.001** |
| Change of fasting glucose (mg/dL)* | -10.15 | 52.74 | -77.18 | 69.73 | **<0.001** | **<0.001** |
| Change of LDL-Cholesterol (mg/dL) | -1.62 | 25.21 | -9.14 | 45.53 | 0.331 | 0.068 |
| Change of HLD-Cholesterol (mg/dL) | 0.88 | 6.28 | 11.00 | 9.93 | **<0.001** | **<0.001** |
| Change of triglycerides (mg/dL)* | -2.50 | 137.15 | -200.11 | 294.82 | **<0.001** | **<0.001** |
| Change of systolic blood pressure (mmHg) | -0.80 | 15.65 | -20.17 | 30.73 | **0.001** | **<0.001** |
| Change of diastolic blood pressure (mmHg) | -3.97 | 10.58 | -13.15 | 19.23 | **0.007** | **<0.001** |
| Change of creatinine (mg/dL)* | 0.61 | 7.10 | -0.03 | 0.14 | 0.123 | 0.870 |
| Change of albumin-creatinine ratio (mg/g)* | 20.25 | 285.83 | -419.98 | 2131.97 | **<0.001** | **0.008** |
| Change of eGFR-MDRD(mL/min/1.73 m^2^) | -3.65 | 13.04 | 1.93 | 12.17 | **0.014** | **0.008** |
| Chang of eGFR-CKD EPI (mL/min/1.73 m^2^) | -4.33 | 15.21 | 2.63 | 14.52 | **0.009** | **0.002** |
| Change of pulse (/min) | 0.87 | 9.80 | -21.08 | 23.25 | **<0.001** | **<0.001** |
| Change of toe tuning fork score, right* | -0.05 | 0.82 | 0.14 | 0.82 | 0.303 | 0.056 |
| Change of toe tuning fork score, left* | 0.00 | 0.82 | 0.14 | 0.80 | 0.400 | **0.050** |
| Change of ankle reflex score, right* | 0.00 | 0.09 | 0.01 | 0.09 | 0.148 | 0.978 |
| Change of ankle reflex score, left* | 0.00 | 0.09 | 0.01 | 0.09 | 0.233 | 0.978 |
| Change of monofilament score, right* | 0.00 | 0.05 | 0.00 | 0.00 | 0.548 | 0.788 |
| Change of monofilament score, left* | 0.00 | 0.08 | 0.00 | 0.00 | 0.935 | 0.594 |
| Change of visual acuity, right* | -0.07 | 0.36 | -0.08 | 0.39 | 0.506 | 0.827 |
| Change of visual acuity, left* | -0.05 | 0.36 | -0.03 | 0.34 | 0.270 | 0.967 |
| Change of non-proliferative retinopathy status, right (%) | 0.07 | 0.50 | 0.00 | 0.56 | 0.581 | 0.772 |
| Change of non-proliferative retinopathy status, left (%) | 0.10 | 0.66 | 0.00 | 0.56 | 0.499 | 0.902 |
| Change of proliferative retinopathy, left (%) | 0.01 | 0.15 | 0.00 | 0.00 | 0.319 | 0.639 |
| Change of proliferative retinopathy (%) | 0.01 | 0.17 | 0.00 | 0.00 | 0.656 | 0.718 |

* Wilcoxon rank-sum test;

** adjusted for age, sex, body mass index, HbA1c, LDL-C, blood pressure, duration of diabetes

Toe tuning fork test: 0 to 8 points when the magnitude of vibrating fork decreased

Ankle reflex score: 0: present; 0.5: present after enhancement; 1: absent

10-g monofilament score: 0: present **(**> 8 points **)**; 0.5: attenuated (1-7 points); 1: absent(0 point)

HbA1c: hemoglobin A1c; LDL: low-density lipoprotein cholesterol; HDL: high-density lipoprotein cholesterol; eGFR MDRD estimated glomerular filtration rate by the Modification of Diet in Renal Disease (MDRD) Study equation; CKD-EPI: Chronic Kidney Disease Epidemiology Collaboration;

**Supplement Table 4. Changes after follow-up for 24 months adjusted by multiple regression**

**in patients with Michigan Neuropathy Screening Instrument Score > 2.5 points**

|  | Medical Therapy | | Bariatric surgery | |  |  |
| --- | --- | --- | --- | --- | --- | --- |
| Number | 42 | | 12 | | P | P** |
| Variable | Mean | StdDev | Mean | StdDev |  |  |
| Change of weight (kg) | -1.74 | 8.93 | -32.22 | 9.91 | **<0.001** | **<0.001** |
| Change of waist circumference (cm) | -3.45 | 12.34 | -27.00 | 9.34 | **<0.001** | **0.002** |
| Change of Body mass index (kg/m^2^) | -0.55 | 3.09 | -11.67 | 4.01 | **<0.001** | **<0.001** |
| Change of HbA1c (%)* | -0.44 | 1.64 | -2.81 | 1.40 | **<0.001** | **0.044** |
| Change of fasting glucose (mg/dL)* | -15.02 | 74.67 | -71.00 | 59.83 | **0.010** | 0.387 |
| Change of LDL-Cholesterol (mg/dL) | -6.79 | 27.18 | -26.90 | 65.55 | 0.365 | 0.210 |
| Change of HLD-Cholesterol (mg/dL) | 8.34 | 56.03 | 13.30 | 15.00 | 0.620 | 0.630 |
| Change of triglycerides (mg/dL)* | -22.81 | 93.36 | -147.00 | 178.12 | **0.013** | **0.033** |
| Change of systolic blood pressure (mmHg) | -2.40 | 13.01 | -15.78 | 26.58 | 0.116 | **0.035** |
| Change of diastolic blood pressure (mmHg) | -4.11 | 8.38 | -11.64 | 11.14 | **0.014** | **0.008** |
| Change of creatinine (mg/dL)* | 0.03 | 0.15 | -0.22 | 0.40 | 0.065 | 0.088 |
| Change of albumin-creatinine ratio (mg/g)* | -11.00 | 123.21 | -75.16 | 365.68 | 0.052 | 0.511 |
| Change of eGFR-MDRD(mL/min/1.73 m^2^) | -1.25 | 8.58 | 10.12 | 23.90 | 0.150 | 0.263 |
| Chang of eGFR-CKD EPI (mL/min/1.73 m^2^) | -2.51 | 11.25 | 14.59 | 27.09 | 0.065 | 0.073 |
| Change of pulse (/min) | 2.79 | 10.58 | -4.56 | 17.64 | 0.200 | 0.511 |
| Change of toe tuning fork score, right* | 0.05 | 0.87 | 0.69 | 0.80 | 0.103 | 0.292 |
| Change of toe tuning fork score, left* | 0.17 | 1.82 | 0.94 | 1.15 | **0.047** | 0.180 |
| Change of ankle reflex score, right* | 0.02 | 0.23 | -0.33 | 0.43 | **0.012** | 0.349 |
| Change of ankle reflex score, left* | 0.00 | 0.29 | -0.33 | 0.43 | **0.016** | 0.757 |
| Change of monofilament score, right* | 0.00 | 0.14 | 0.06 | 0.17 | 0.361 | 0.479 |
| Change of monofilament score, left* | 0.00 | 0.14 | 0.06 | 0.17 | 0.351 | 0.481 |
| Change of visual acuity, right* | -0.13 | 0.26 | -0.02 | 0.19 | 0.402 | 0.892 |
| Change of visual acuity, left* | -0.13 | 0.36 | -0.25 | 0.21 | 0.125 | 0.193 |
| Change of non-proliferative retinopathy status, right (%) | 0.17 | 0.49 | 0.40 | 0.55 | 0.368 | 0.806 |
| Change of non-proliferative retinopathy status, left (%) | 0.14 | 0.47 | 0.40 | 0.55 | 0.279 | 0.879 |
| Change of proliferative retinopathy, left (%) | 0.00 | 0.31 | 0.00 | 0.00 | 1.000 | 0.622 |
| Change of proliferative retinopathy (%) | 0.00 | 0.32 | 0.00 | 0.00 | 1.000 | 0.614 |

* Wilcoxon rank-sum test;

** adjusted for age, sex, body mass index, HbA1c, LDL-C, blood pressure, duration of diabetes

Toe tuning fork test: 0 to 8 points when the magnitude of vibrating fork decreased

Ankle reflex score: 0: present; 0.5: present after enhancement; 1: absent

10-g monofilament score: 0: present **(**> 8 points **)**; 0.5: attenuated (1-7 points); 1: absent(0 point)

HbA1c: hemoglobin A1c; LDL: low-density lipoprotein cholesterol; HDL: high-density lipoprotein cholesterol; eGFR MDRD estimated glomerular filtration rate by the Modification of Diet in Renal Disease (MDRD) Study equation; CKD-EPI: Chronic Kidney Disease Epidemiology Collaboration;
